# Supplementary material for: Targeted proteomics of plasma extracellular vesicles uncovers MUC1 as combinatorial biomarker for the early detection of high-grade serous ovarian cancer
Source: J Ovarian Res. 2024 Jul 17;17:149. doi: 10.1186/s13048-024-01471-8 (PMC11253408; doi:10.1186/s13048-024-01471-8)
Supplement: Supplementary file 1 — Supplementary Material 1. [file 13048_2024_1471_MOESM1_ESM.docx]

**Targeted Proteomics of Plasma Extracellular Vesicles Uncovers MUC1 as Combinatorial Biomarker for the Early Detection of High-grade Serous Ovarian Cancer**

**Tyler T. Cooper^1,2^, Dylan Z. Dieters-Castator^3^, Jiahui Liu^4^, Gabrielle M. Siegers^4^, Desmond Pink^4^, Lorena Veliz^5^, John D. Lewis^4^, Yangxin Fu^4^, François Lagugné-Labarthet^5^, Helen Steed^6^, Gilles A. Lajoie^1†^, and Lynne-Marie Postovit^2,4,6†.^**

1Department of Biochemistry, Western University, London ON, Canada;

2Department of Biomedical and Molecular Sciences, Queen’s University, Kingston ON, Canada;

3Department of Anatomy and Cell Biology, Western University, London, ON, Canada;

4Department of Chemistry, Western University, London, ON, Canada;

5Department of Oncology, University of Alberta, Edmonton AB, Canada;

6Department of Obstetrics and Gynecology, University of Alberta, Edmonton AB, Canada;

†To whom correspondence should be addressed.

E-mail: l.postovit@queensu.ca

Date: June 10^th^, 2024


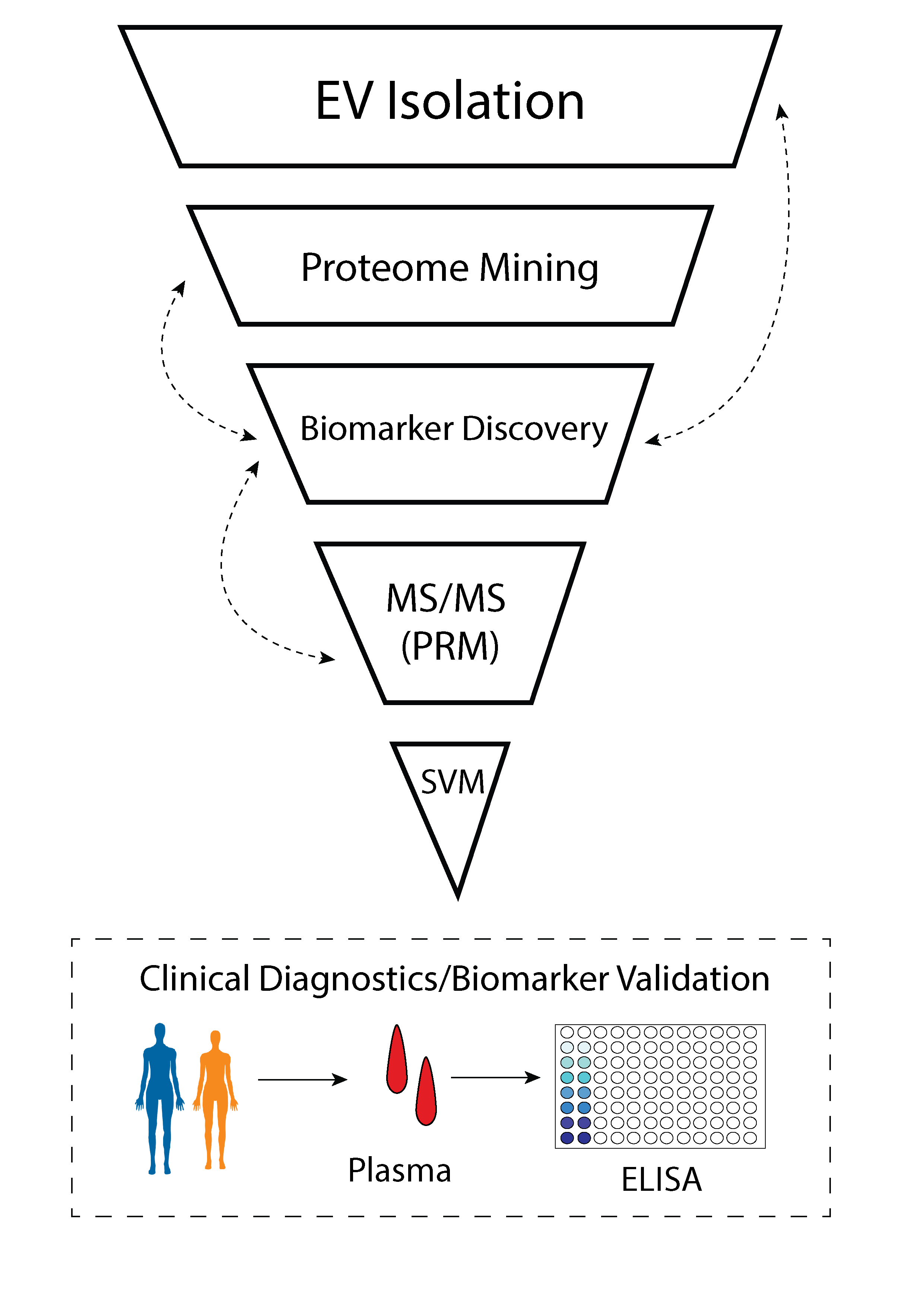


Graphical Abstract

**
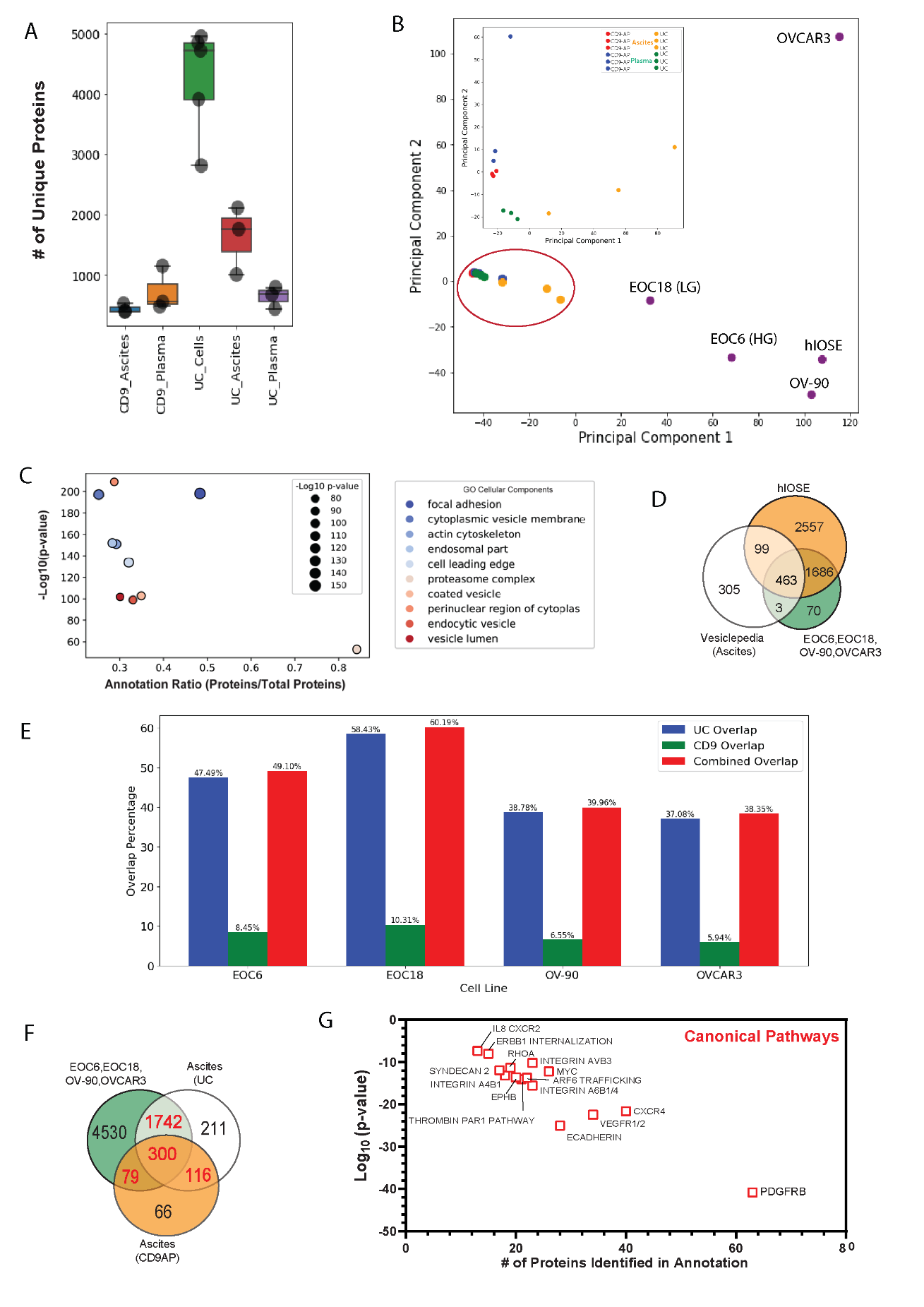
**

**Supplemental Figure 1. Proteomic analysis of cell line, plasma, ascites-derived EVs.** A) Number of unique proteins identified in plasma, ascites or cell line EVs isolated by either ultracentrifugation (UC) or CD9 affinity purification. B) Principle component analysis of biofluid vs cell line EVs. Insert shows only biofluid samples and segregation based on isolation method. C) GO cellular component enrichement analysis (GOCC) of proteins identifed in cell line EVs. D) Comparison of cell EV proteomes to Vesiclepedia database filtered for ascites fluid. E-F) Overlap of protein identified in cell line EVs compared to ascites EVs isolated by either UC or CD9 affinity purification. G) Cannocial pathway enrichment analysis of protein common between cell line and ascites EVs.

**
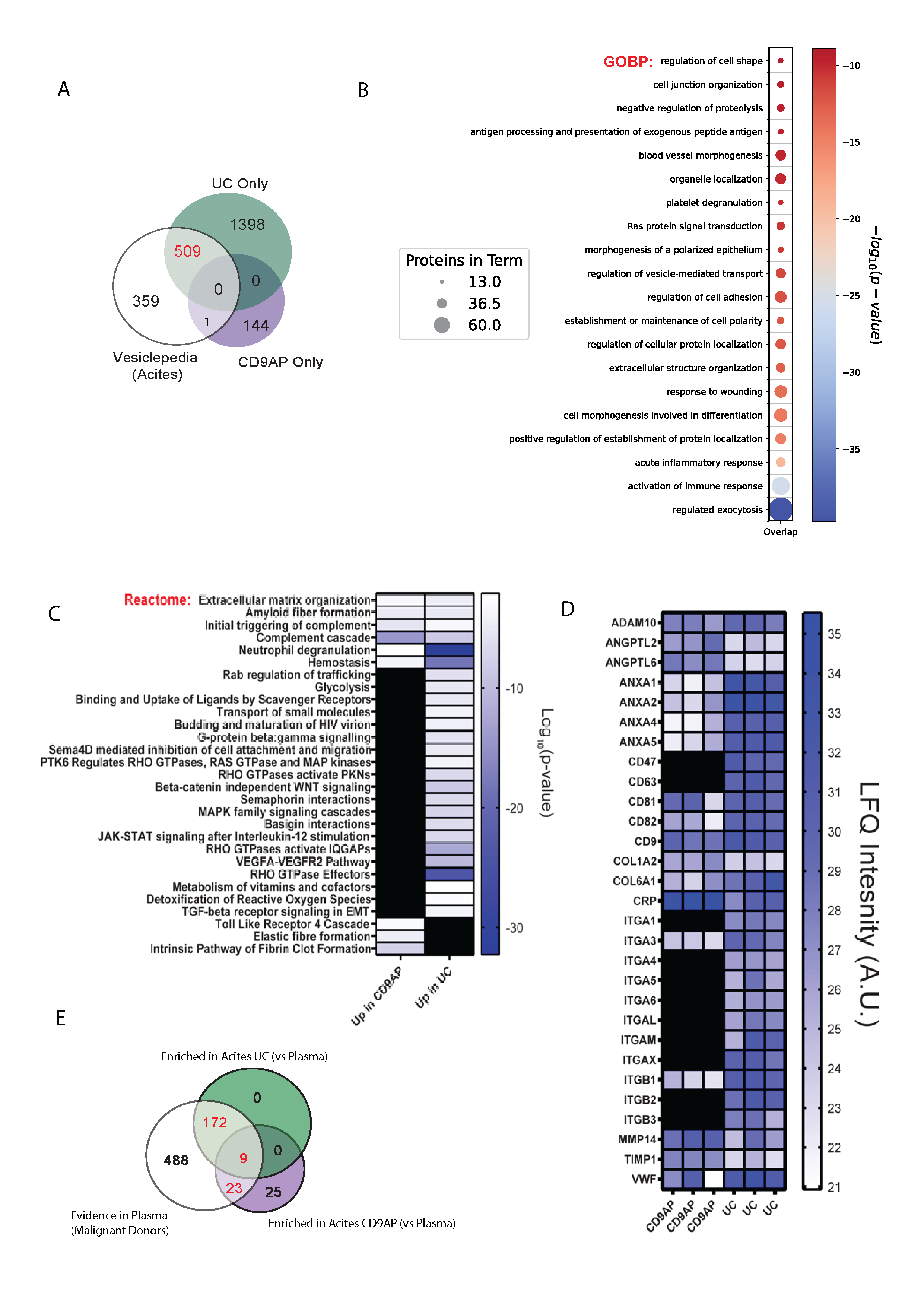
**

**Supplemental Figure 2. Mining the protome of biofuid EVs isolated by UC or CD9 affinity purification.** A) Comparision of proteomes for ascites EV isolated by UC vs CD9-AP vs Vesiclepedia database filtered for ascites fluid. B) GO Biological enrichement analysis of proteomes obtained from UC and CD9-AP isolated EVs. C) Reactome enrichement analysis of proteins signficantly enriched in UC-isoalted ascites EVs compared to CD9-AP isolated EVs. D) Common EV proteins detected in ascites EVs isoalted by UC or CD9-AP. E) Venn diagram of protein eriched in ascites fluid EVs compared to plasma EVs that were detected in plasma of patients with HGSC.

**
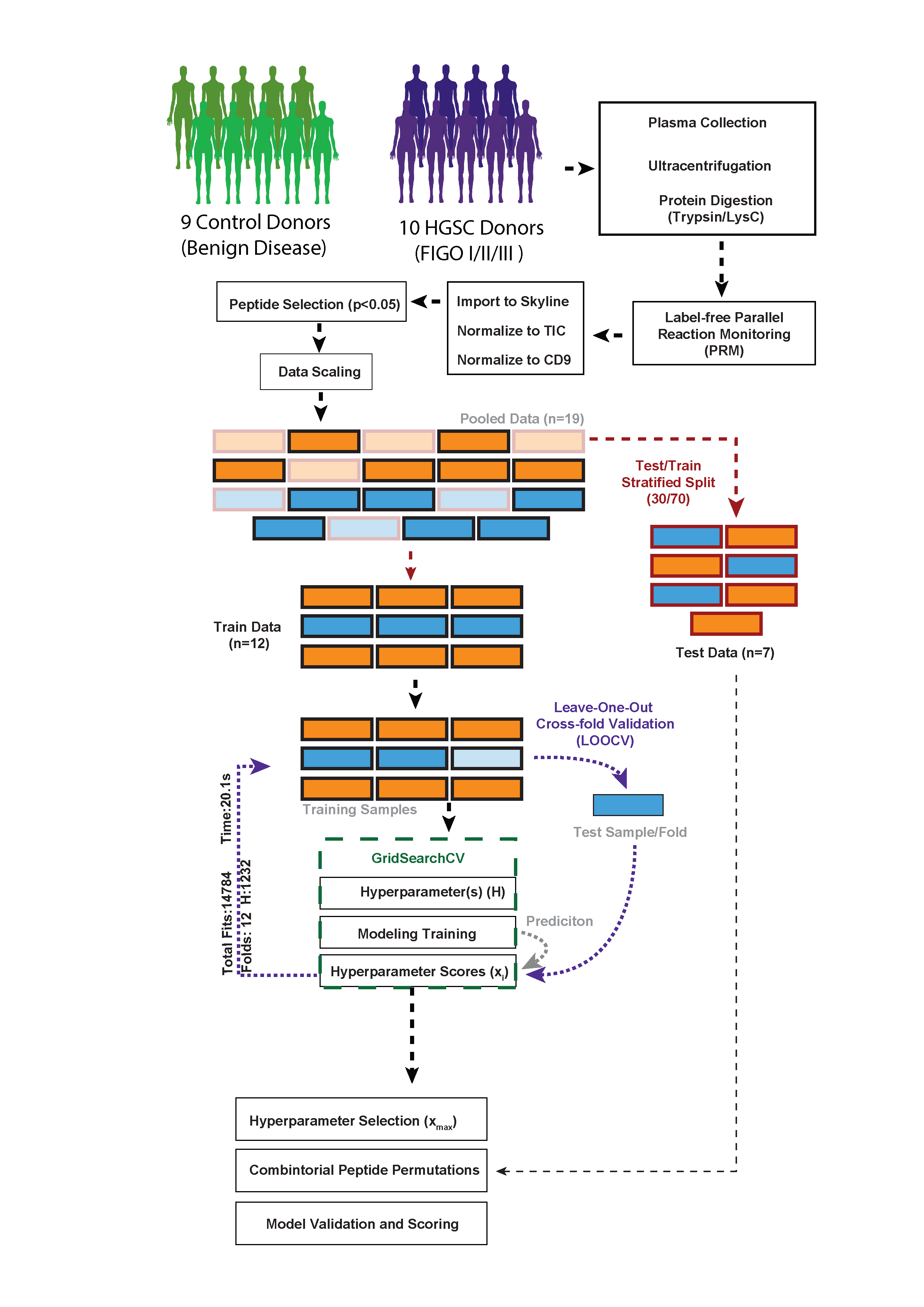
**

**Supplemental Figure 3. Overview of biomarker discovery pipeline using Edmonton plasma cohort.**

**
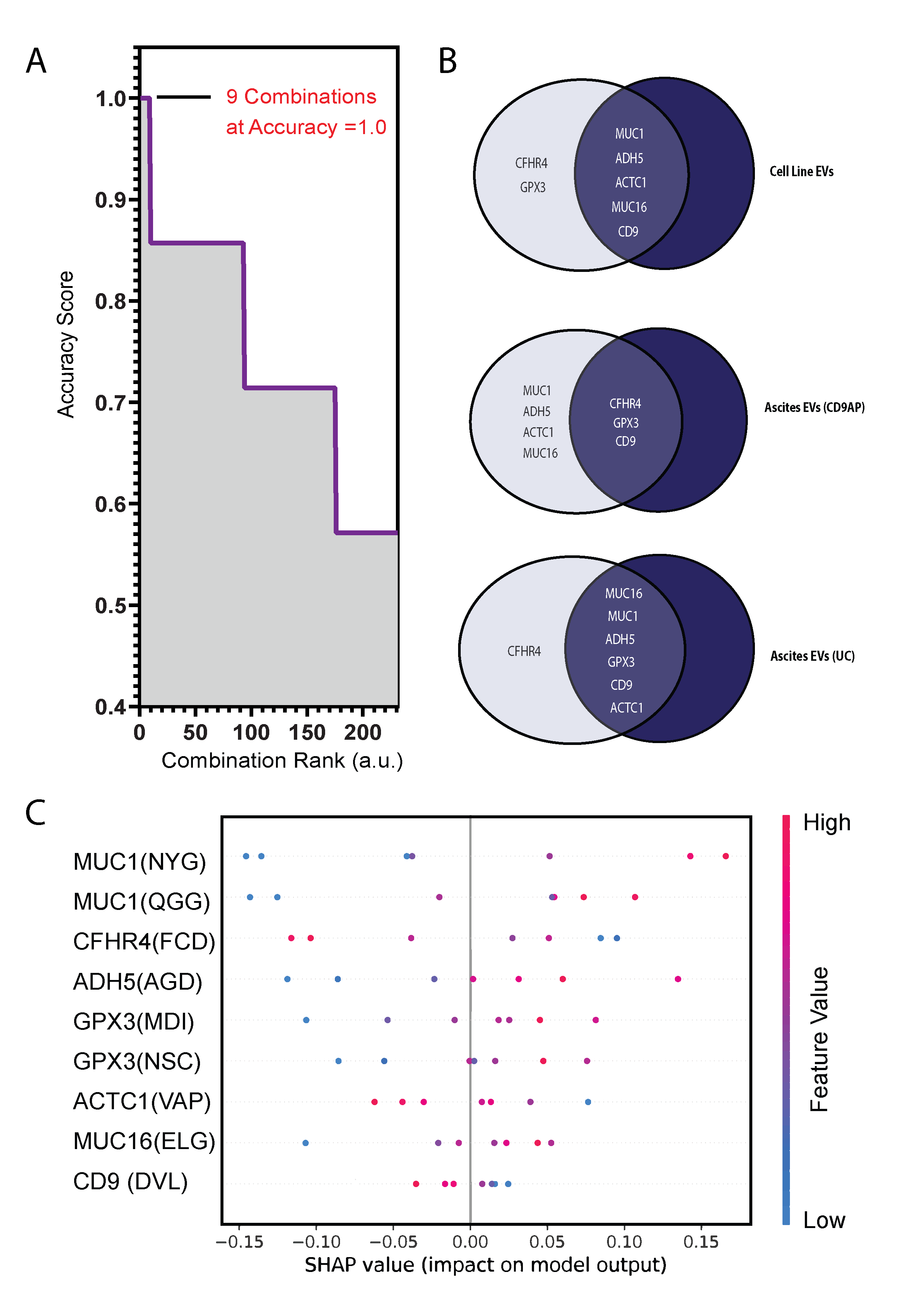
**

**Supplemental Figure 4.** Summary of support vector machine training and analysis using the Edmonton cohort. A) 9 SVM models were found to have ROC-AUC = 1.0 when validated with test data set. B) Retrospective assesement using evnn diagrams to highlight source and methodology of protein identification for initial spectral library generation. C) SHAPley model analysis. Positve SHAP values indiccate a feature was important in classifying HGSC donors.

**
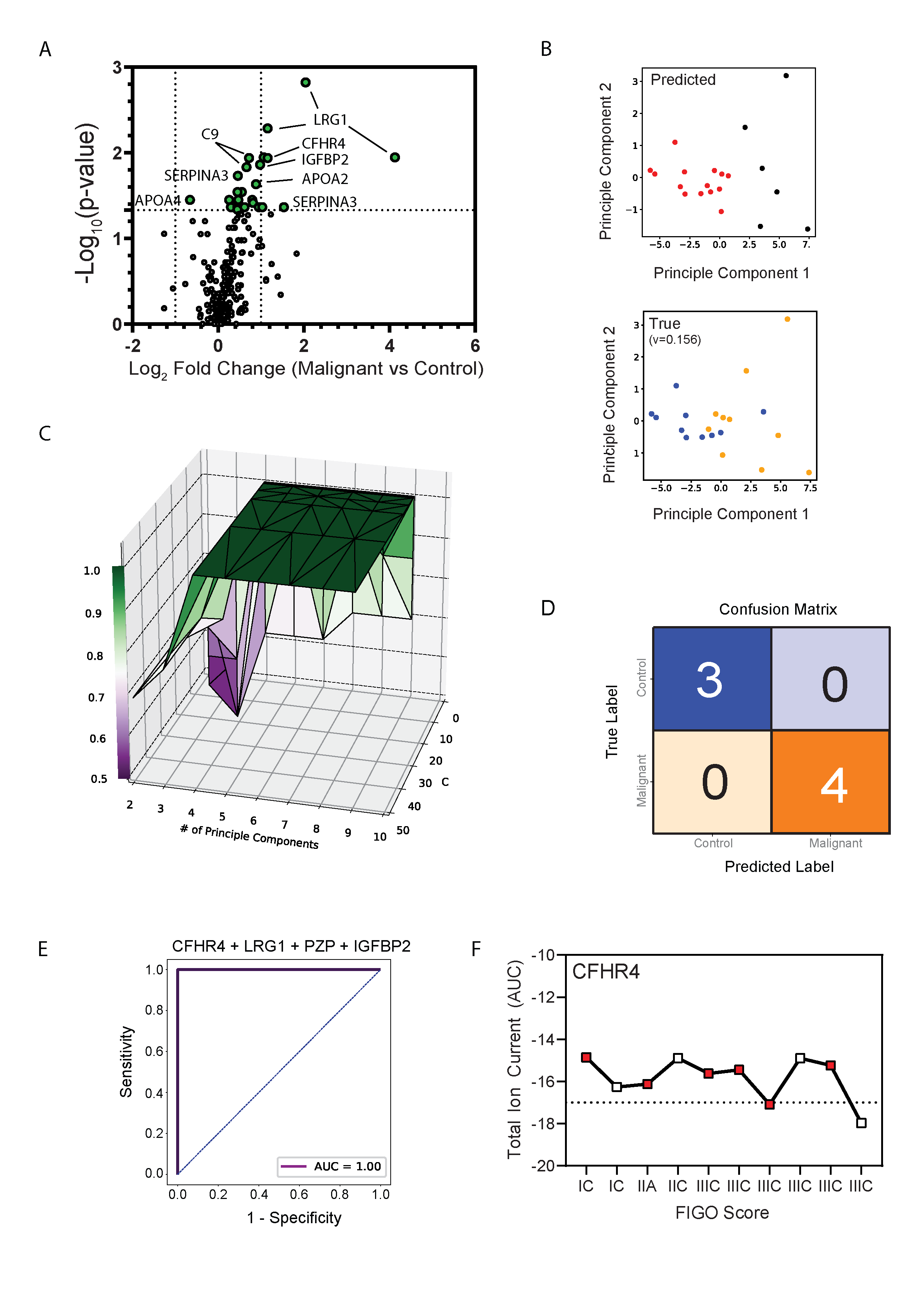
**

**Supplemental Figure 5.** PRM and Support Vector Machine analysis of EV-depleted plasma from Edmonton cohort. A) Volcano plot showing differential enrichment proteins between HGSC and benign controls. B) PCA projection of data with k-means clustering showing predicted (top) and true (bottom) labels. Benign is represented as red and blue, whereas HGSC is represented as black and orange. C) Summary of SMV model training. D-E) Confusion matrix and ROC-AUC curve of SVM prediction usng 4-protein signature. F) Normalized ion current for CFHR4 in training (red) and test (white) data.

**
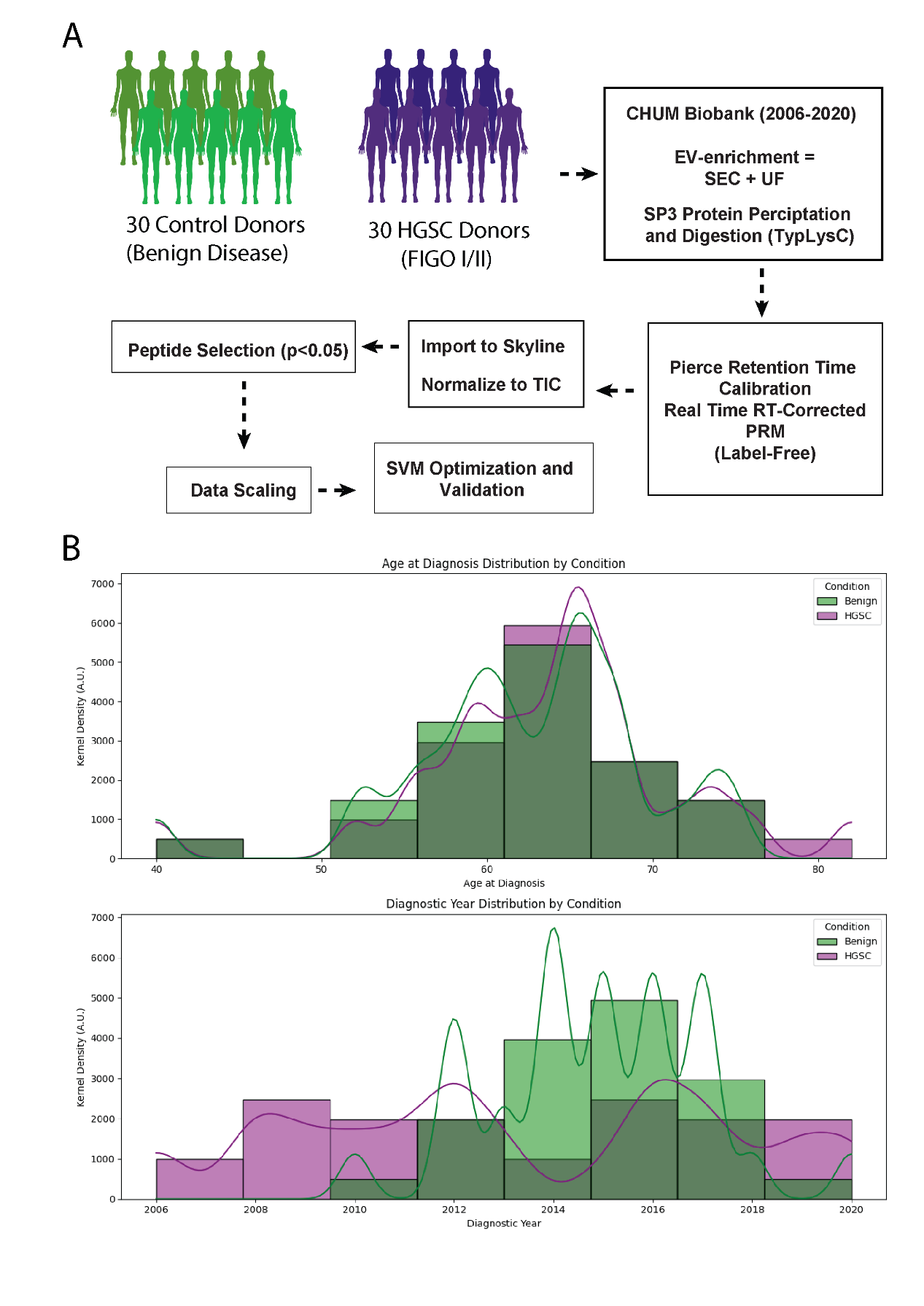
**

**Supplemental Figure 6. Summary of biomarker discovery in CRCHUM cohort.** A) Summary of workflow using plasma obtained from CRCHUM. B) Characteristics of plasma donors.

**
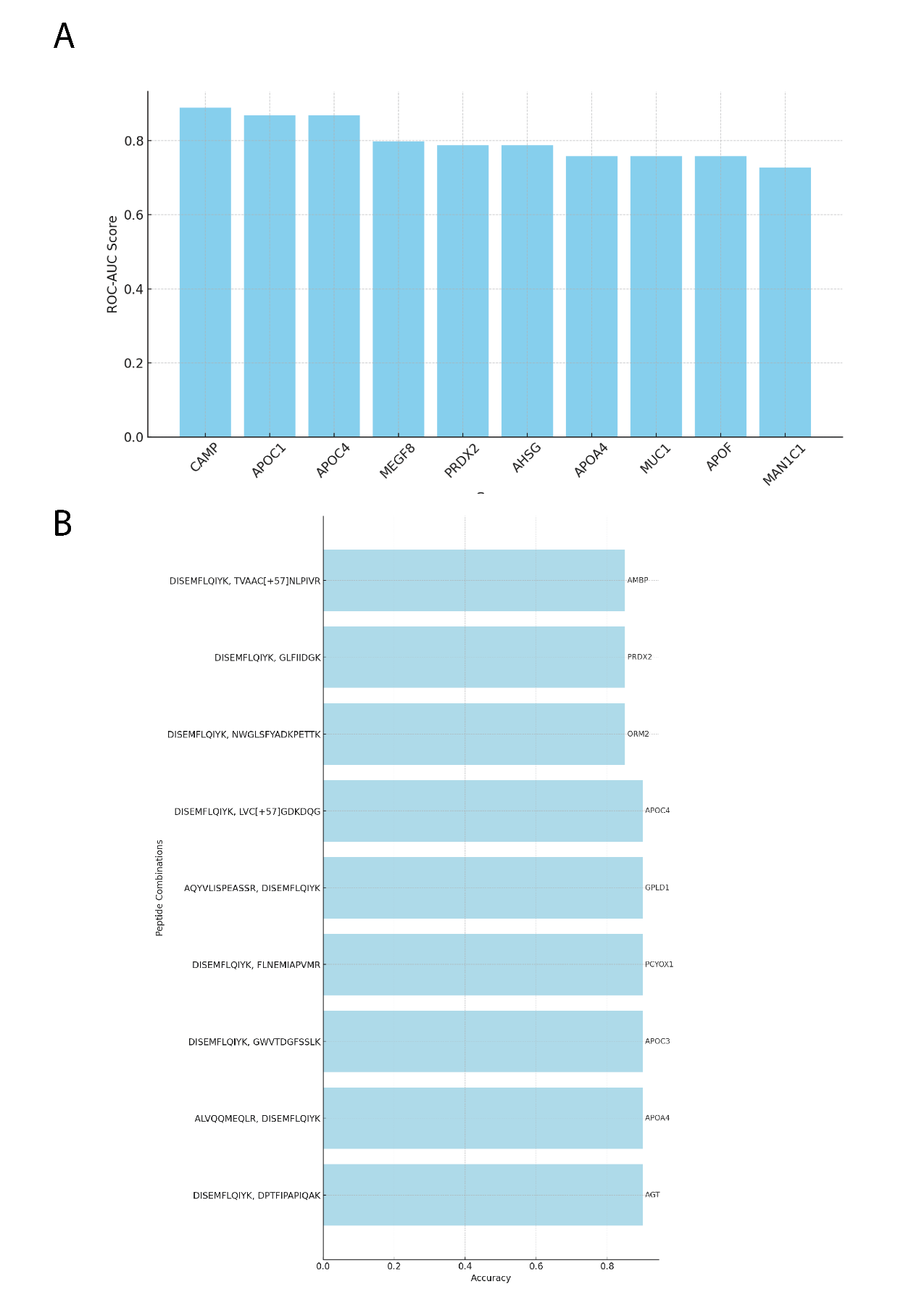
**

**Supplemental Figure 7. MUC1 is a combintorial biomarker.** A) ROC-AUC of proteins as stand alone biomarker using logistic regression. B) MUC1 (DISEMFLQIYK) provides high accuracy when used in combination with other biomakers.

**Supplemental Table 1.** Q Exactive (Plus) instrument parameters for data acquisition.


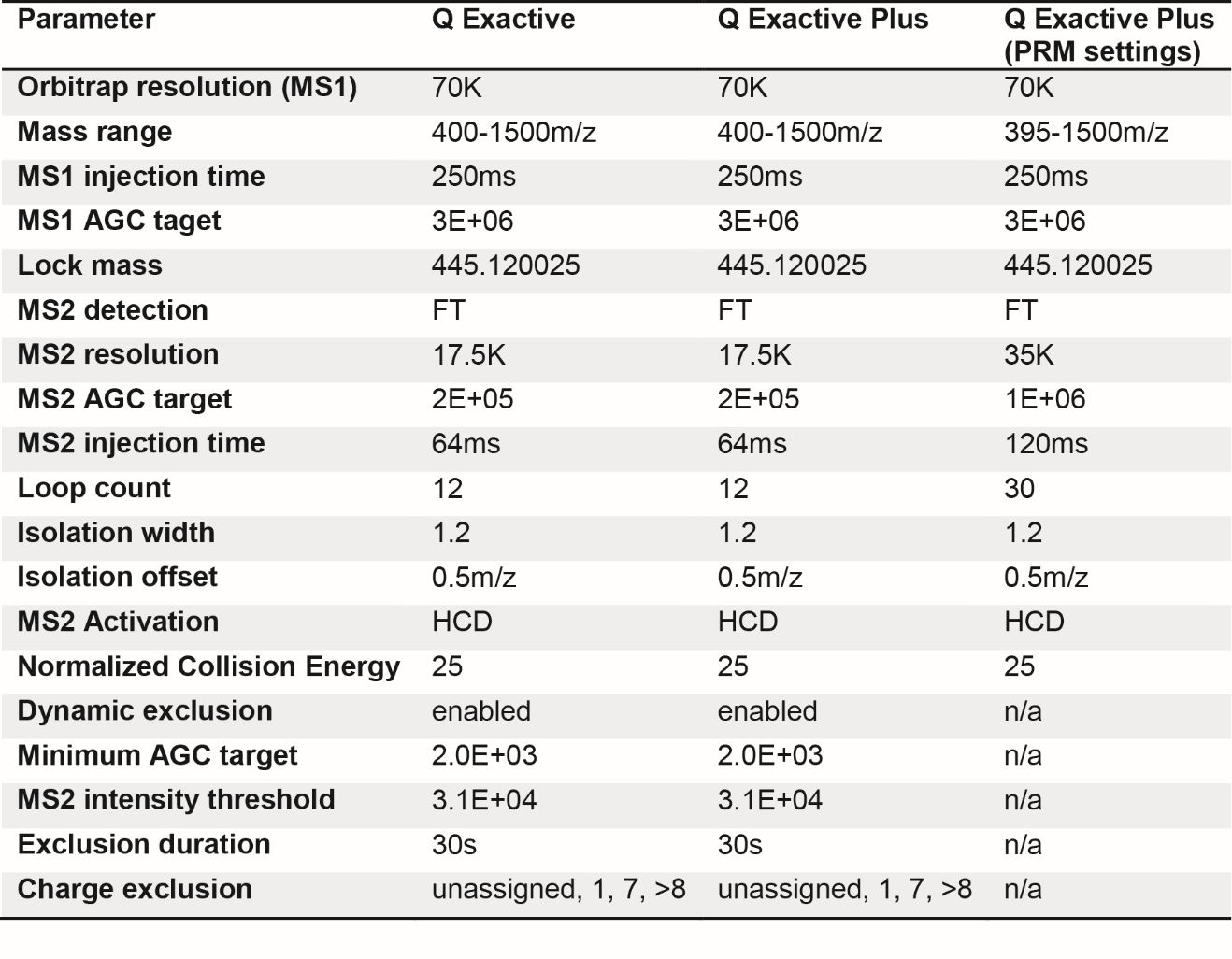


**Supplemental Table 2. Eclipse Instrument Settings for GPF-DIA Library, GPF-DIA Acquisition, and PRM.**

| Parameter | GPF-DIA Library | GPF-DIA Sample | PRM |
| --- | --- | --- | --- |
| MS1 Resolution | **60K** | **60K** | **120K** |
| MS1 Scan Range | **400-1000** | **400-1000** | **400-1000** |
| MS1 AGCTarget | **4E5** | **4E5** | **4E5** |
| MS1 Max IT | **50 ms** | **50 ms** | **Auto** |
| MS2 Resolution | **30K** | **30K** | **60K** |
| MS2 Scan Range | **Auto** | **Auto** | **200-1800** |
| MS2 AGC Target | **1E5** | **1E5** | **1E5** |
| Iso. Window (m/z) | **50 x 4 m/z (50%)** | **50 x 24 m/z (50%)** | **1.2** |
| NCE | **33** | **33** | **33** |
| MS2 Max IT | **54 ms** | **54 ms** | **118 ms** |
| Dynamic RT | **N/A** | **N/A** | **Pierce PRTC Mixture** |

**Supplemental Table 3.** Nanoparticle Tracking Analysis on Ascites EVs isolated by Ultracentrifugation or CD9 Immunopurification.

| Parameter | UC-EVs | CD9AP-EVs |
| --- | --- | --- |
| Concentration (particles/mL) | 4.32x10^8^ ± 3.74x10^7^ | 6.07 x 10^8^ ± 1.37 x 10^8^ |
| Particles/frame | 21.9 ± 1.9 | 33.4 ± 7.5 |
| Completed tracks (Sum) | 8042 | 16057 |
| Mean | 266.6 ± 14.8 | 106.4 ± 10.5 |
| Mode | 183.5 ± 13.5 | 81.3 ± 3 |
| D10 | 153.7 ± 9.9 | 62.1 ± 1.5 |
| D50 | 204.1 ± 15.1 | 85.5 ± 5.8 |
| D90 | 519.4 ± 5.8 | 166.2 ± 27.6 |

**Supplemental Table 4.** Patient characteristics of HGSC samples used for PRM analysis.

| Number of samples | 10 |
| --- | --- |
|  |  |
| Age at diagnosis |  |
| Mean | 54.8 |
| Median | 54 |
| Range | 39-69 |
|  |  |
| FIGO stage |  |
| IC | 2 |
| IIA | 1 |
| IIC | 1 |
| IIIC | 6 |
|  |  |
| Alive | 5 |
| Deceased | 5 |

**Supplemental Table 5.** **EV-enriched Blood Plasma Peptides Selected for Targeted Proteomics and SVM model optimization.**

| **Peptide** | **Gene** | **Log_2_ Fold Change**  **(M vs. C)** | **-Log_10_**  **(p-value)** | **SVM Model Relevance**  **(% of SVM Model)** |
| --- | --- | --- | --- | --- |
| MDILSYMR | GPX3 | 1.31 | 2.25 | 33% |
| QGGFLGLSNIK | MUC1 | 2.57 | 2.03 | 33% |
| FCDMPVFENSR | CHFR4 | 1.06 | 1.89 | 33% |
| YVPPSSTDR | MUC1 | 3.11 | 2.70 | 22% |
| AGDTVIPLYIPQCGECK | ADH5 | -1.20 | 1.40 | 22% |
| DVLETFTVK | CD9 | -0.24 | 1.34 | 22% |
| NSCPPTSELLGTSDR | GPX3 | 0.98 | 1.61 | 11% |
| ELGPYTLDR | MUC16 | 2.12 | 1.22 | 11% |
| VAPEEHPTLLTEAPLNPK | ACTC1 | -0.98 | 2.21 | 0% |
| NYGQLDIFPAR | MUC1 | 1.98 | 2.08 | 0% |
| IISIMDEK | PZP | 1.80 | 2.04 | 0% |
| AYAAGFGDR | TNC | 1.74 | 1.76 | 0% |
| LDAPSQIEVK | TNC | 1.61 | 1.76 | 0% |
| VISQIAMNDEK | SLC34A2 | 2.29 | 1.66 | 0% |
| FEIENCLANK | PZP | 1.47 | 1.66 | 0% |
| ASSFLGEK | C4B | 1.89 | 1.65 | 0% |
| AYSLFSYNTQGR | APCS | 0.89 | 1.56 | 0% |
| EDSPFALK | PZP | 1.66 | 1.55 | 0% |
| ATAQMLEVMFK | LBP | 0.91 | 1.46 | 0% |
| SIPQVSPVR | CPN1 | 0.60 | 1.45 | 0% |
| VATYLPAPEGLK | TNC | 1.61 | 1.36 | 0% |
| DNELLVYK | APCS | 0.83 | 1.34 | 0% |

**M = Malignant, C=Control, SVM= Support Vector Machine, *Linear SVM (C=0.025)**

**Supplemental Table 6.** Patient characteristic of CHUM Samples PRM samples

| Number of samples | 60 |
| --- | --- |
|  |  |
| Age at diagnosis |  |
| Mean | 62.8 |
| Median | 64 |
| Range | 40-82 |
|  |  |
| FIGO stage |  |
| IA | 7 |
| IB | 1 |
| IC | 8 |
| IIA | 6 |
| IIB | 7 |
| IIC | 1 |
